# Parents’ Perceptions Regarding the Implementation of a Physical Therapy Stimulation Program for Children with Disabilities in Bolivia: A Qualitative Study

**DOI:** 10.3390/ijerph17176409

**Published:** 2020-09-03

**Authors:** Sagrario Pérez-de la Cruz, Ivonne Ramírez

**Affiliations:** 1 Department of Nursery, Physiotherapy and Medicine, University of Almería, La Cañada de San Urbano, 04120 Almería, Spain; 2 Instituto de Investigaciones en Neurodesarrollo, University of San Francisco Xavier de Chuquisaca, 212 Zona Central, Sucre, Bolivia; ramirez.ivonne@usfx.bo

**Keywords:** disability, parents, children, physical therapy, Bolivia

## Abstract

The purpose of this study was to explore how parents of children with neuromotor disorders in the department of Chuquisaca (Bolivia) perceive attendance to a physical therapy stimulation program and the expectations they place on the therapy and professional care provided to their children. Semi-structured interviews were conducted with the parents, related to their role in supporting the recommended exercise program for the child, generating topics such as benefits of the therapy for the child, impact on the family, and role of the project in terms of therapy and the physical therapists providing treatment, including both positive and negative aspects of the overall process. This study revealed the importance of understanding the feelings of families receiving intervention under a pioneering program in Bolivia for the detection and treatment of children with neuromotor disorders. Being able to access these types of services provides them with extensive personal, social, and economic support. Knowing their concerns, desires, and demands will allow us to continue to improve and offer the best care for children and families. The professionals involved should also be encouraged to develop effective teaching techniques to promote the inclusion of parents in the stimulation program.

## 1. Introduction

The birth of a child with a disability is an event that has a major impact on the entire family [1]. Overall, the family tries to cope with this adverse event by embarking on the arduous task of finding new therapeutic options to minimize the future effects of the disability on the child. Parents/caregivers often assume the main responsibilities for the long-term care of these children [2,3].

The prevalence of cerebral palsy (CP) in the Plurinational State of Bolivia is 0.24 per 1000 inhabitants, which increases to 0.52 per 1000 when considering the population under 15 years of age, lower than that described in Western countries, which ranges from one to five cases per 1000 inhabitants [4,5]. However, exact data on the general population and, more specifically, on the population of children with CP, the most frequent types of disabilities, and the results of functional evaluations are unknown, as there is a lack of reliable population records [6].

Culturally, in South America, and particularly in Bolivia, a relative is expected to take responsibility for the care of the person with a disability. Parents represent the usual caregivers, and they are expected to accept or assume this role regardless of the possible emotional, physical, and financial burden involved. In addition, this entails a situation where parents are unprepared to deal with this situation, including scenarios where future plans are broken, income-generating activities in the family are abandoned due to having to take care of the child during the entire day, and there is decreased free time and greater susceptibility for a decline in the health status of the child and parents [7]. Furthermore, the caregivers/parents’ abilities to maintain their own emotional, personal, physical, social, and financial well-being may be seriously compromised [7,8].

Quantitative research identified a number of possible barriers to treatment adherence in children with disabilities. These include the complexity of the prescribed regimen, parents’ limited knowledge of the proposed therapy for their child, and relationships and interactions with health professionals [9,10]. However, there is a recognized need for qualitative studies to understand the complexities of this situation. Knowing these experiences could improve the chances of caring for the emotional interactions between family members of children with disabilities and the professionals who work in direct contact with them [4]. Several qualitative studies evaluated factors that influence the adherence to treatment of children with disabilities [11,12]; however, very few of these investigated the influence that attendance to therapy has on the parents of these children and the perceived benefits or needs of the parents [13]. Furthermore, there is a dearth of studies conducted in the socio-cultural context of Latin America. Thus, to date, there are no known studies in Bolivia that focus on the impact of caring for children with disabilities. As a result, the first-hand perceptions of parents/caregivers of these children within the Bolivian culture are being overlooked.

The present study was designed to examine the perceptions of parents attending therapy with their children in the Department of Chuquisaca (Bolivia) and to understand the impact of attending a therapeutic stimulation program focused on covering the neuro-psychomotor needs of children.

## 2. Materials and Methods

This study is part of the research project 2016DEC018, funded by the Andalusian Agency for International Cooperation of the Government of Andalusia (Spain), and it was approved by the Bioethics Committee of the University of Almeria.

### 2.1. Design

This is a qualitative study, using an interpretive phenomenological approach. The qualitative design of this study included focus groups, considering that group interactions can trigger responses and generate ideas that cannot emerge during interviews [14]. The use of interviews within focus groups was employed in other studies to identify experiences related to parental adherence to proposed treatments. The reason for grouping the parents was to facilitate parents’ collaboration by providing them with the ability to attend these groups during the same timetable as their child’s treatment.

### 2.2. Participants

This study included parents of physically disabled children who were attending rehabilitation sessions in the departmental program for the detection and care of children with alterations in their neuro-psychomotor development carried out in the Department of Chuquisaca (Bolivia). Participants were parents of children who attended the care program for neuro-psychosensory risk, funded by the Andalusian Agency for International Development Cooperation, University of Almeria (Spain) in collaboration with the University of San Francisco Xavier de Chuquisaca (Bolivia). They attended face-to-face sessions with physical therapists, where they received sessions, and the payment was symbolic (as they were families with limited economic resources). This was agreed upon so that the economic cost would not be a reason for them to abandon treatment. The children belonged to the city of Sucre and the Department of Chuquisaca. Thus, the inclusion criteria were parents/guardians of children aged 0–5 years, who were prescribed an intervention program by a physical therapist in the program. Subjects were excluded from the study if they presented communication problems (communication in a language other than Spanish, such as Quechua), failure to understand the issues raised, and failure to regularly attend sessions.

A purposive sampling strategy was used to include parents/guardians of children across different age groups, genders, and clinical conditions. Although the sample size depended on information saturation, 27 subjects were initially selected (all of whom were women, as they were the only ones attending the care programs). A professional from each center sent a letter to parents who met the indicated criteria to invite them to participate in the focus group discussion. Ten days later, the mothers were contacted by telephone (since they were the only ones willing to participate) to determine their participation and to clarify any doubts that might have been raised. The distribution of the sample in focus groups was performed with the criterion of geographical location. Since there were several locations where the program was implemented, each one was formed with the mothers who attended the program.

### 2.3. Data Collection

The interviews were conducted over a four-week period (November 2019). The interview was previously scripted by the interviewer (I.R.). An audio recording was made of each of the interviews at the headquarters. The interviews lasted 20–35 min. Notes were taken of the actions and attitudes observed during the course of the interview. The interviews were then transcribed verbatim.

### 2.4. Ethical Considerations

All participants were requested to grant informed consent before the start of the study, informing the participants of the purpose of the study. This consent was signed by each of the mothers who wanted to participate in the study. In addition, the integrity and confidentiality of all data obtained throughout the study were assured, as stated in the Personal Data Protection Act of 1999 [15]. The anonymity of the participants was preserved. Participants were informed of the entire research process, and their privacy was protected, especially their thoughts and beliefs, as well as the privacy of the recordings, which were kept in custody and inaccessible to people external to the study.

### 2.5. Data Analysis

Two researchers, unknown to the parents, carried out the distribution of the interviews, with the help of a topic guide with predetermined questions (Table 1). This guide was based on a review of the literature in this area carried out by those in charge of providing therapy at each of the sites. Additional questions were included, depending on the topics chosen, which arose from an initial focus group [16]. The interviews were audio- and video-recorded, and field notes were used for data collection.

Table 1 displays the themes and subthemes used during the interviews with the participating mothers.

A thorough verbatim transcript of each of the interviews, field notes, and other emerging details was used to compile the documents, to enable their qualitative analysis. In order to include personal documents in the analysis, authenticity (generated within the context), credibility (produced by the interviewer), representativeness (description of personal experiences), and meaning (providing information relevant to the study) were firstly verified [16].

The findings obtained were integrated separately into the same analysis matrix. Subsequently, they were combined in joint meetings, where data collection and analysis procedures were discussed. In the case of differences of opinion, the identification of the problem was carried out by consensus among the team members. All researchers participated in the coding and analysis; however, the task was supervised by researchers with experience in qualitative research (I.R. and S.P.-d.l.C.). No data analysis software was used.

## 3. Findings

### 3.1. Participants

**Figure 1:**
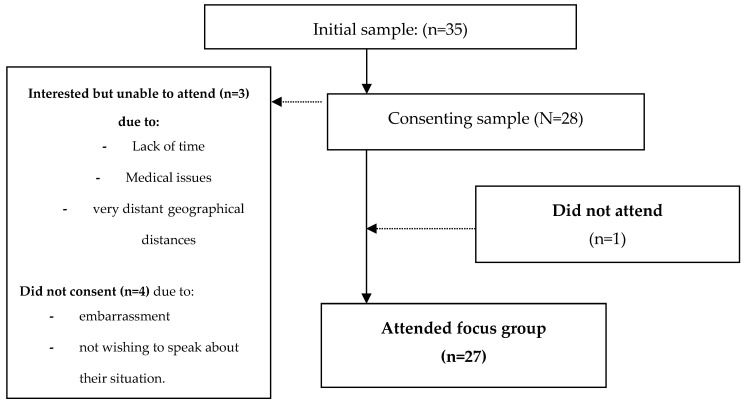
Recruitment process and study participation.

The final sample consisted of 27 mothers, based on an initial sample of 35 eligible participants/families attending the program. The mean age of the children attending therapy was 2.8 years (the program included care for children 0–5 years of age). Figure 1 shows the process of recruitment and study participation. The reasons for non-attendance and/or dropout were unknown. The non-participants displayed a similar profile to participants.

The socio-demographic characteristics of the participants in the study are shown in Table 2.

Participants reported that their children received one to four weekly sessions, consisting of 30 to 45 min of treatment at the centers. Treatment programs varied among the children, with Vojta therapy sessions (for children 0–2 years old) and Bobath therapy and psychomotor sessions for children over that age. The participants of the treatment program for children with disabilities acknowledged experiencing difficulties in continuing therapy for long periods of time, with adherence to the program and attendance being an important issue for them.

The analysis revealed both positive and negative aspects of the perception of mothers of children with different neurodevelopmental disorders. This analysis provided information concerning the perception of the therapy and the perception of the project as such. As for the subthemes, the benefits for the child, the impact on the family, and other aspects related to the staff and administrative issues were highlighted, as outlined in Table 3.

The parents’ perceptions of the therapy program are reflected in the following adjectives used to describe the program: “necessary and a great help for the child and the family” (mother of a child with cerebral palsy), “a new opportunity” (mother of a child with cerebral palsy), and “grateful” (mother of a child with developmental disabilities). This was used as a way to open the interviews; afterward, further in-depth questions ensued regarding more specific aspects of the study.

### 3.2. Positive Aspects of Therapy Reported by Mothers

#### 3.2.1. Participation

The participation of the mothers was high, because a large part of the group was formed of housewives, who have occasional work or are self-employed. This explains the high attendance of mothers during the 1–2-h period of the intervention.

“I come to see what they do because, for me, it is important for the rehabilitation of my daughter; in the time I spent in the project she improved a lot…”

(*Mother of a child with cerebral palsy*)

“I stay with her during therapy, so I can learn and apply it at home; I see what they do with her and how my daughter progresses.”

(*Mother of a child with Down syndrome*)

Many of the mothers, due to their low level of education, did not get involved and remained seated close to their children. Very few managed to understand the treatment and, above all, weighed up the results they noticed during the sessions.

“… I come to watch and to be able to help him at home; that would be very important for us—for them to teach the parents exercise techniques to be able to practice at home.”

(*Mother of a child with cerebral palsy*)

#### 3.2.2. Trust and Understanding

The respondents demonstrated a high level of confidence and trust in the treatment, which was strengthened by noting the improvements in their children.

“The changes became apparent throughout this process, and we are more attentive and willing to help with the treatment that therapists provide.”

(*Mother of a child with cerebral palsy*)

“I see that he is improving, and, over time, the changes are noticeable; they make him work longer, they do a lot of exercises, and he is active.”

(*Mother of a child with cerebral palsy*)

Most mothers joined the project after previous experiences attending other centers in the city, which failed to meet their expectations because of the poor progress the children made in their development. By observing objective improvements in their child’s motor, social, language, and cognitive development, the mothers expressed confidence in the treatment the children were receiving, expecting to see further progress in the future.

#### 3.2.3. Benefits of Therapy for the Child

This is another factor that strengthens confidence in therapy, i.e., the child’s achievements, supporting adherence to treatment as noticed by mothers, who see their effort rewarded because of the motor, language, socialization, and cognitive progress.

In all cases, positive changes were observed that were more objective in cases of neuromotor impairment and more subjective in cases of autism spectrum disorder (ASD). However, the latter changes are more valued by mothers who suffer rejection because of the difficulty in educational inclusion due to the low socialization and language development of their children.

“There are improvements in the movement of his feet and hands; little by little, he is raising his head; before he wasn’t even able to keep his head up; he was lying on the bed and just looking at the ceiling.”

(*Mother of a child with Down syndrome*)

“My son is improving a lot; we can keep still now; before it was impossible; we didn’t know how to handle him, and I always hoped that he would call me mom, that he would be a child that would communicate, but he is like a little baby; he still wears a diaper.”

(*Mother of a child with ASD and attention deficit and hyperactivity disorder (ADHD)*)

“My tears dried up when I got here; he’s firmer, he sits alone longer, he doesn’t bite his little fingers so often anymore… he can now integrate with his classmates, he listens to the teacher, and he is more connected to what the other children are doing, even though he doesn’t play with them yet.”

(*Mother of a child with psychomotor delay*)

“We’ve been coming here for three years. I’ve had a lot of help during this time. In just one year, they managed to make my daughter walk; before that, I felt discouraged and I didn’t know where to go… or what to do…”

(*Mother of a child with cerebral palsy*)

#### 3.2.4. Improvements and Benefits for the Family

Favorable changes can be seen in the family dynamic, because of the links that are strengthened with the other members by the child’s achievements and because he or she is already a more active subject in the functioning of the nucleus as such. The opinion of the family (father and siblings, above all) plays an important role in supporting the mother’s adherence to therapy.

“The child shows an interest in communicating and interacting. His dad is more attentive to my son; occasionally, he accompanies us to therapy.”

(*Mother of child with ASD*)

“When we consistently attend therapy, yes, it helps him with concentration and he manages to pay attention to us; his dad almost didn’t believe in the treatment, but little by little he began to see results.”

(*Mother of a child with global developmental delay*)

“Yes, now he gets along better with family members. All is well; there is a good interaction between siblings.”

(*Mother of a child with cerebral palsy*)

#### 3.2.5. Attention by Staff

Regarding the attention provided by the physical therapy staff, the mothers compared their attendance to previous public programs where their children received therapy, stating that there was no saturation of patients, the treatment was personalized and kind and, therefore, parents valued the warmth of the personnel attending their children.

“The treatment that the therapists give us is very good; they are all good; they are always aware of what we need.”

(*Mother of a child with cerebral palsy*)

“As for the therapists, we have no complaints; they treat us well, ask questions, and talk to us. Sometimes they call me if we are missing sessions because we don’t have money to come by public transport.”

(*Mother of a child with cerebral palsy*)

“All the little doctors are very nice—all of them, the graduates and the students too. They are very good; they give us all their attention, to all the things they see as progress; they are always asking.”

(*Mother of child with ASD*)

### 3.3. Negative Aspects of Therapy Reported by Mothers

With regard to the weaknesses perceived by the mothers, two categories were extracted: information and training given to the mothers of children with disabilities, and administrative issues. The hours restricted to the working hours of university institutions stood out, which interfered with the working hours of the parents, creating low satisfaction in this aspect in a minority group, given that the largest group constituted housewife mothers who have greater ease in adapting.

“Longer hours, we can’t coincide; since I work, I have no one to bring him in and that hurts us and makes the child miss his therapies.”

(*Mother of a child with Down syndrome*)

Another aspect perceived as negative was the modification of therapeutic schemes, namely, uncoordinated methodologies and therapies for each child, given that there are several therapists, and these can rotate when treating the children. There is little information provided to parents when the working plans are modified, and this point is very much related to the continuous turnover of therapists by the university institution.

“The fact that students rotate and don’t work for long periods of time, come in and out of classes, do not always treat for the full hour; it would be good if they didn’t have different therapists, but perhaps assign only one each year.”

(*Mother of a child with cerebral palsy*)

“More coordination among therapists, with the exercises they do, to improve the children’s condition; often they are starting a treatment, they change the therapist, and once again they change the therapy.”

(*Mother of child with ASD*)

#### Training

An important point was the training and information they expect to receive from the project. They considered this aspect very important in order to support their children at home and continue the improvement in the future.

“More communication about treatment so that we can do it at home.”

(*Mother of a child with cerebral palsy*)

“Training parents to be able to work with our children at home.”

(*Mother of child with ASD*)

“That they can teach parents exercise techniques to be able to implement at home.”

(*Mother of child with ASD*)

“That they explain to us in terms we can understand what our child’s diagnosis is and how we can intervene.”

(*Mother of a child with global developmental delay*)

## 4. Discussion

This is a pioneering study in Latin America, given that, when reviewing the existing literature, there are currently no studies of this type in Latin American society that reflect this problem from the point of view of caregivers of children with any type of disability. References were found in other countries [17,18,19,20] such as Cambodia [19] (whose level of development may be similar to the situation in Bolivia, although they are culturally very different). Therefore, the findings obtained in the former study should not be extrapolated to Bolivian society. Another study carried out in the Canary Islands (Spain) [20] reflects a similar situation. However, based on the interviews conducted, a plausible explanation for similar findings may be the similarity of the social environment in which the child’s daily life takes place.

This study supports previous research showing that children with or without risk of developmental disorders are susceptible to low adherence to exercise prescriptions to be performed in the home [21,22]. The findings of these studies indicate that the characteristics of the home exercise program and the physical therapist’s teaching methodology may influence adherence. Culturally, a purely medical model is in place in Bolivia, where the patient (in this case, the parents of the affected child) does not take an active role in the child’s recovery, leaving decisions regarding treatment proposals and execution of therapies to the physician. Studies such as those carried out by Conde [23] and Venturiello [24] refer to the passive role that male fathers play in the therapy of their children and how female mothers are the ones who work through the initial shock and disbelief more quickly, interposing favorable resources such as seeking help, investigating specific facilities, persevering with treatment programs, collaborating with therapies, and taking charge of their child’s therapeutic process. The mothers participating in our study demonstrated their ability to participate in the therapy applied to their children, to understand what is being done and the methodology used, and to be able to reinforce the improvements attained by attending therapy sessions, as long as the professionals showed confidence in a mutual collaboration between both parties. This study provides knowledge for professionals in considering the tasks and strategies that can be used to develop and implement a home exercise program, with adequate levels of adherence, adapted to the social–cultural level of the parents.

Although it is the mothers who seek treatment and are the driving force behind a new therapeutic alternative, the positive changes in the child’s development encourage the fathers to become involved, accompanying, supporting, and even approving the child’s continued attendance.

Another positive aspect of this project was that parents highlighted feelings of confidence in the performance of the therapy in each of the children attending the program. Three factors were particularly important in creating a climate of trust: regular contact with parents/caregivers, providing information on the pathology, and evolution and response provided in therapy. This action by the professionals led to improvements described by the parents in their child’s health, not only on a physical level, but also on a cognitive, social, and family level. A positive result of this climate of cooperation is the low percentage of abandonment that occurred in the program, even when the families were not candidates for regular attendance due to economic or geographical reasons. All of these advantageous situations were reflected in similar studies that were carried out with the opinions of parents with children with disabilities [25,26,27,28]. Therefore, the inclusion of parents of patients in the therapy administered, by providing advice, home management assistance, progressions, and a diversity of therapeutic alternatives, is an important objective to be pursued [28,29,30].

This study showed that most parents highly valued the experience of the early intervention team. They specified the contribution that the professionals made to their prospects for improvement and/or modification of the pathology, and they encouraged activities to build on the accomplishments made in therapy. In addition, the parents themselves suggested the incorporation of exercises for home or training in the management of their children, to reinforce therapy within their daily routine and to overcome any challenges that may arise when doing so. These statements are related to qualitative documents on adherence, on the basis of children with chronic conditions [31]. According to some authors, parents’ knowledge of physical and cognitive improvements may have a paradoxical effect; thus, parents’ perception of outcomes in children often encourages them to continue with treatment, whereas, on the other hand, it may cause parents to relax and decrease their adherence [30,32,33]. In our case, attendance was regular—without prolonged or unjustified absences—and was not altered by the children’s progress. On the contrary, since families have been attending therapy, their life (both the child’s and the family’s) has undergone positive changes. Nonetheless, they would like therapy to take up less of their time, due to work and family obligations that alter their daily dynamics, and due to the economic cost of this type of service in countries where there is no possibility of continuous and accessible treatment.

This study also identified factors that professional physical therapy staff could use to try to improve adherence, for example, development of individualized programs for each family (responding to their socio-cultural peculiarities), respecting their preferences, resources, and daily routines. Physical therapists must be sensitive to the detection of the needs of each family unit at each stage of development to facilitate adherence, despite the changes involved in the arrival of a child with special needs [34,35]. During the first treatment session, physical therapists should also maintain frequent contact with parents to review their professional performance and provide feedback to build trust [36]. The findings also suggest that professionals should strive to develop effective teaching techniques to promote active parental involvement in their child’s treatment program. More research is needed in this regard, as well as research on factors related to parents, because the findings were mainly limited to factors related to therapy and the professionals caring for their children that contribute to poor treatment adherence. Several factors were identified during the interviews that could be helpful for professionals to recognize early signs of non-adherence to therapy. Examples of these factors include concern about the correct prescription of therapy, fear of causing harm, difficulty incorporating exercise into daily routines, boredom/tiredness/apathy on behalf of parents, and feelings of uncertainty. Therapists should be alert to any warning signs that may appear throughout the treatment, especially in the case of younger and more affected children, due to the extended duration of interventions, as well as at the onset of treatment.

### Limitations

Longitudinal and prospective studies would be appropriate involving children with different pathologies or deficits in order to evaluate the entire rehabilitation process. Another limitation is that this is a systematic analysis of the responses received, without having divided these into subgroups (for example, by gender, age, economic level, etc.) and the fact that, during communication with parents attending the treatment program, some of the potential participants were unable to communicate fluently in Spanish. Hence, it would have been interesting to be able to collect further information from these parents. Given the size of the sample, caution is required when generalizing the findings obtained.

## 5. Conclusions

Despite the small sample, the current study revealed the importance of understanding the sentiments of the families who are receiving intervention under a pioneering program in Bolivia for the detection and treatment of children with neuro-motor disorders. A child with special needs entails a number of demands for the family at the economic, effective, social, and relational levels. Being able to access these types of services provides extensive support for the parents of these children. By knowing their concerns, desires, and demands, we can continue to improve and offer the best care for children and families. In turn, future studies should examine what structural changes could be made to increase parent satisfaction and maintain attendance in the proposed program. Physical therapists should be encouraged to develop effective teaching techniques to promote the inclusion of parents in the child care program.

## Figures and Tables

**Table 1:** Summary of study themes, subthemes, and categories.

Theme	Subtheme	Categories
Parents’ perceptions of therapy	Benefits of therapy on the child	Parental involvement Trust in the therapy Understanding and learning Motor progress Changes in socialization Language development Effects on cognition
	Impact on the family	Changes in the family dynamic Involvement Effects on other areas
Perception of the project from the parents’ perspective	Role of the project in terms of therapy and staff	Perception of positive changes in child development Lack of time
	Personal	High level of satisfaction and good treatment on behalf of the staff Poor coordination in methodologies

**Table 2:** Data from the study sample (*n* = 27).

(a) Age of mother			
18–25 years	26–35 years	36–45 years	45–50 years
6 (22.22%)	16 (59.25%)	4 (14.81%)	1 (3.7%)
(b) Educational level of mothers			
No studies	Basic education	Secondary education	Professional
8 (29.62%)	7 (25.92%)	9 (33.33%)	3 (11.11%)
(c) Socio-economic level			
Very low	Low	Mid-level	High
5 (18.51%)	17 (62.96%)	4 (14.81%)	1 (3.7%)
(d) Type of child pathology			
Motor pathology of neurological origin	Global development delay	Down syndrome	Other neurodevelopmental disorders
17 (62.96%)	5 (18.51%)	1 (3.7%)	4 (14.81%)

**Table 3:** Parents’ perceptions of therapy (*n* = 27).

(a) Positive aspects of therapy noted by parents	
Parent attendance at therapy	21 (77.77%)
Understanding treatment	23 (85.18%)
Trust in therapy	26 (96.29%)
Attention by staff	21 (77.77%)
Demand for training and information	18 (66.66%)
Overall satisfaction with care provided	17 (62.96%)
(b) Negative aspects of the project noted by parents	
Restricted schedules	6 (22.22%)
Poor staff coordination for therapies	4 (14.81%)
Staff turnover	3 (11.11%)
Specific methodologies and therapies	3 (11.11%)
